# Supplementary material for: Age-related demethylation of the TDP-43 autoregulatory region in the human motor cortex
Source: Commun Biol. 2021 Sep 21;4:1107. doi: 10.1038/s42003-021-02621-0 (PMC8455575; doi:10.1038/s42003-021-02621-0)
Supplement: Supplementary file 2 — Supplementary information [file 42003_2021_2621_MOESM2_ESM.pdf]

Supplementary information

Age-related demethylation of the TDP-43 autoregulatory region in the human motor cortex

Yuka Koike[1], Akihiro Sugai[2], Norikazu Hara[3], Junko Ito[4], Akio Yokoseki[5], Tomohiko Ishihara[1], Takuma Yamagishi[1], Shintaro Tsuboguchi[1], Mari Tada[6], Takeshi Ikeuchi[3], Akiyoshi Kakita[4], Osamu Onodera[1]

[1] Department of Neurology, Brain Research Institute, Niigata University, Niigata city, Japan

[2] Department of Molecular Neuroscience, Center for Bioresource-based Research, Brain Research Institute, Niigata University, Niigata city, Japan

[3] Department of Molecular Genetics, Center for Bioresource-based Research, Brain Research Institute, Niigata University, Niigata city, Japan

[4] Department of Pathology, Brain Research Institute, Niigata University, Niigata city, Japan

[5] Department of Inter-Organ Communication Research, Niigata University Graduate School of Medical and Dental Sciences, Niigata city, Japan

[6] Department of Pathology Neuroscience, Center for Bioresource-based Research, Brain Research Institute, Niigata University, Niigata city, Japan

Corresponding author: Akihiro Sugai and Osamu Onodera

E-mail: akihiro.sugai@bri.niigata-u.ac.jp; onodera@bri.niigata-u.ac.jp

1-757 Asahimachi-dori, Chuo-Ku, Niigata-City, Niigata 951-8585, Japan

Tel. and Fax: +81 25 227 0663

Supplementary Fig. 1

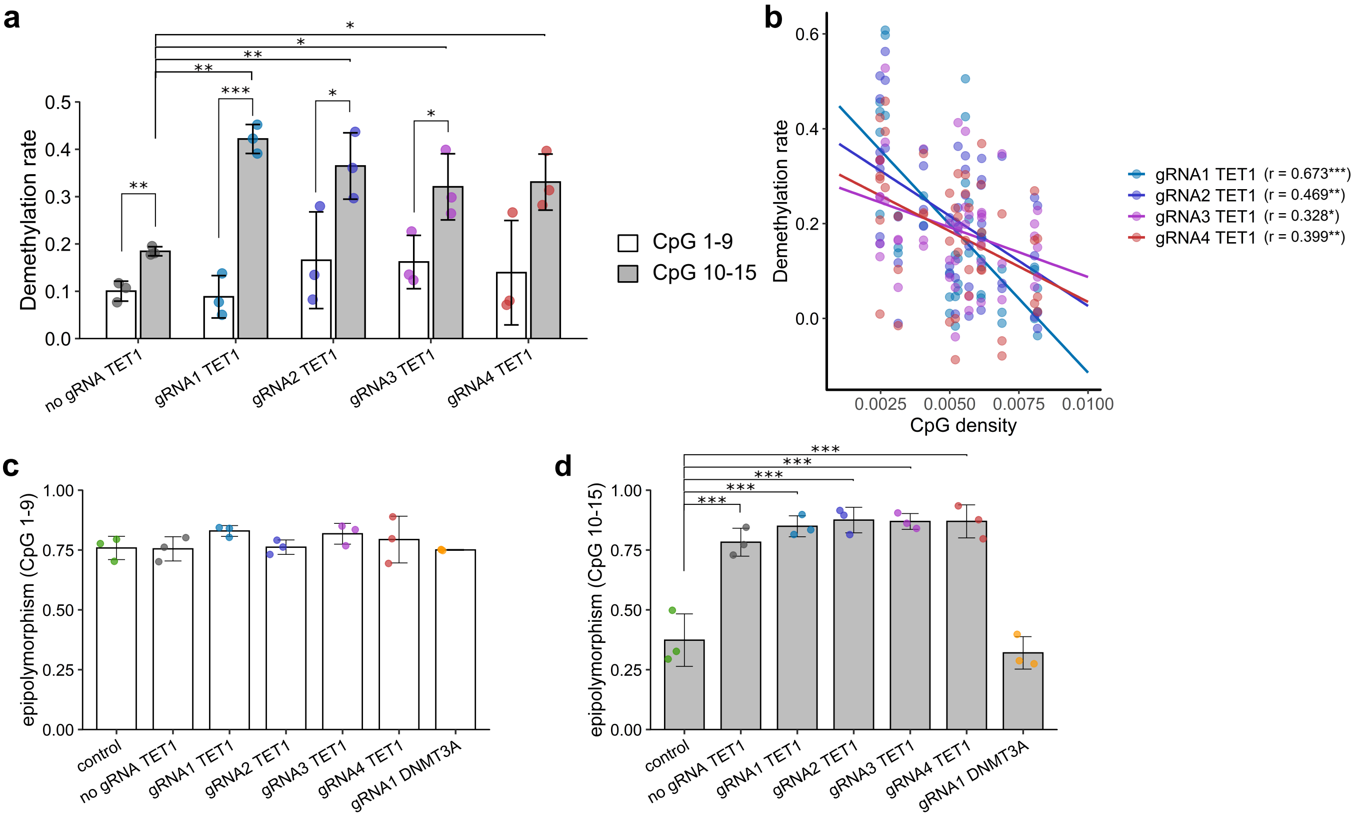

Supplementary Fig. 1 Demethylation with the TET1-TARDBP-target vector at 3'UTR CpGs 1–15. (a) Demethylation rate in the controls at CpGs 1–9 and CpGs 10–15 (mean±SD, n=3). Student's t-test; comparison of CpGs 1–9 and CpGs 10–15. Dunnett's test; each guide RNA for no guide RNA. (b) Correlation between the demethylation rate and the CpG density at each CpG site. Pearson's correlation test. (c)(d) Epipolymorphism scores of CpGs 1–9 and CpGs 10–15. Dunnett's test relative to the controls. *p<0.05, **p<0.01, ****p<0.001.

Supplementary Fig. 2

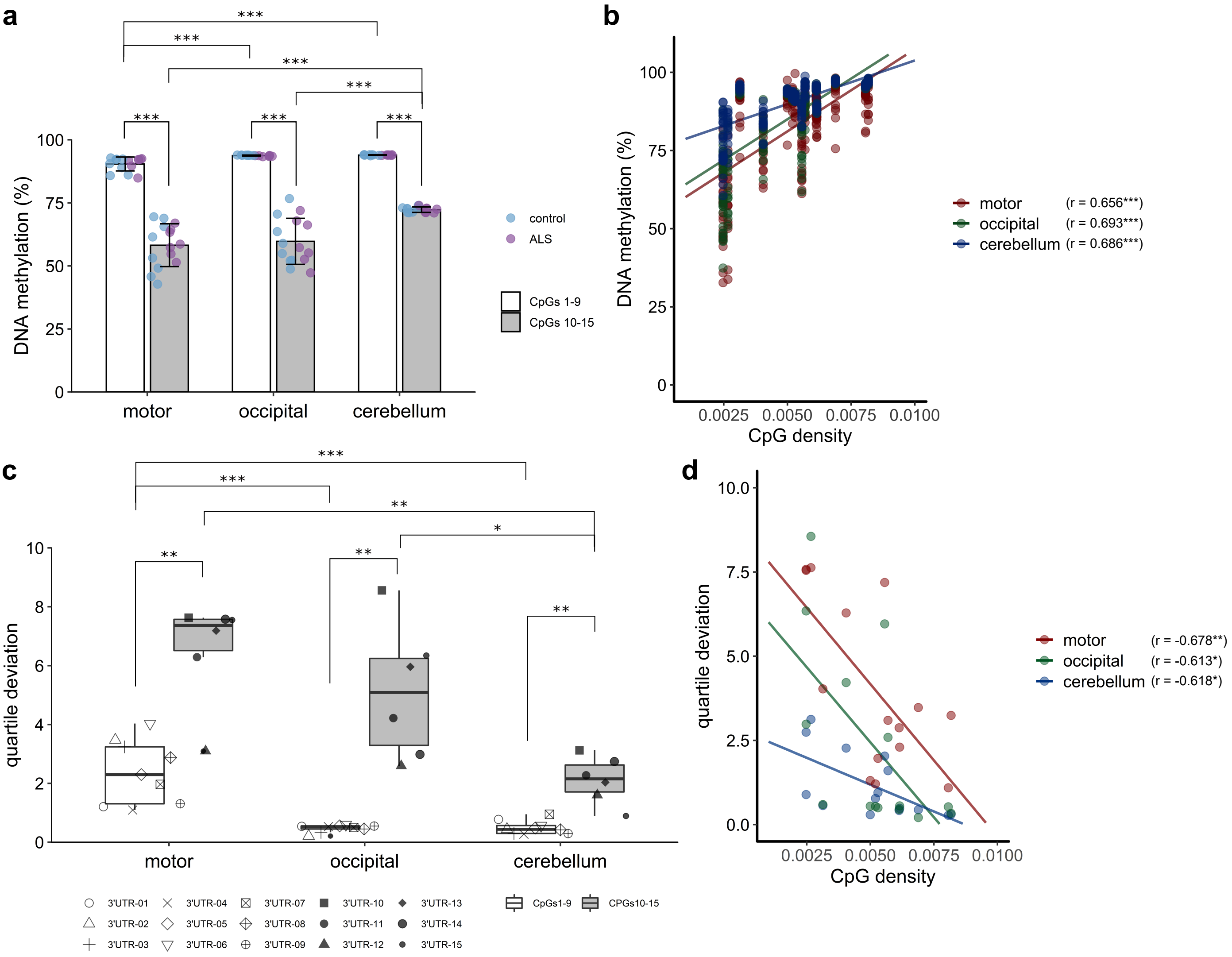

Supplementary Fig. 2 DNA methylation of the TARDBP gene in the human brain. (a) Mean DNA methylation percentages of 3'UTR CpGs 1–9 and 3'UTR CpGs 10–15 (mean±SD). (b) Correlation between the percent DNA methylation and the CpG density at each CpG site. (c) Quartile deviation of the percentage of DNA methylation at each CpG site. Box plots of 3'UTR CpGs 1–9 and 3'UTR CpGs 10–15 in each brain region are shown; Welch's t-test was used for the comparisons between 3'UTR CpGs 1–9 and 3'UTR CpGs 10–15; Bonferroni's multiple comparison test was used for the comparisons across the brain regions. (d) Correlation between the quartile deviation of the percentage of DNA methylation and the CpG density at each CpG site. Pearson's correlation test. Seven ALS patients and 8 controls. *p<0.05, **p<0.01, ****p<0.001.

Supplementary Fig. 3

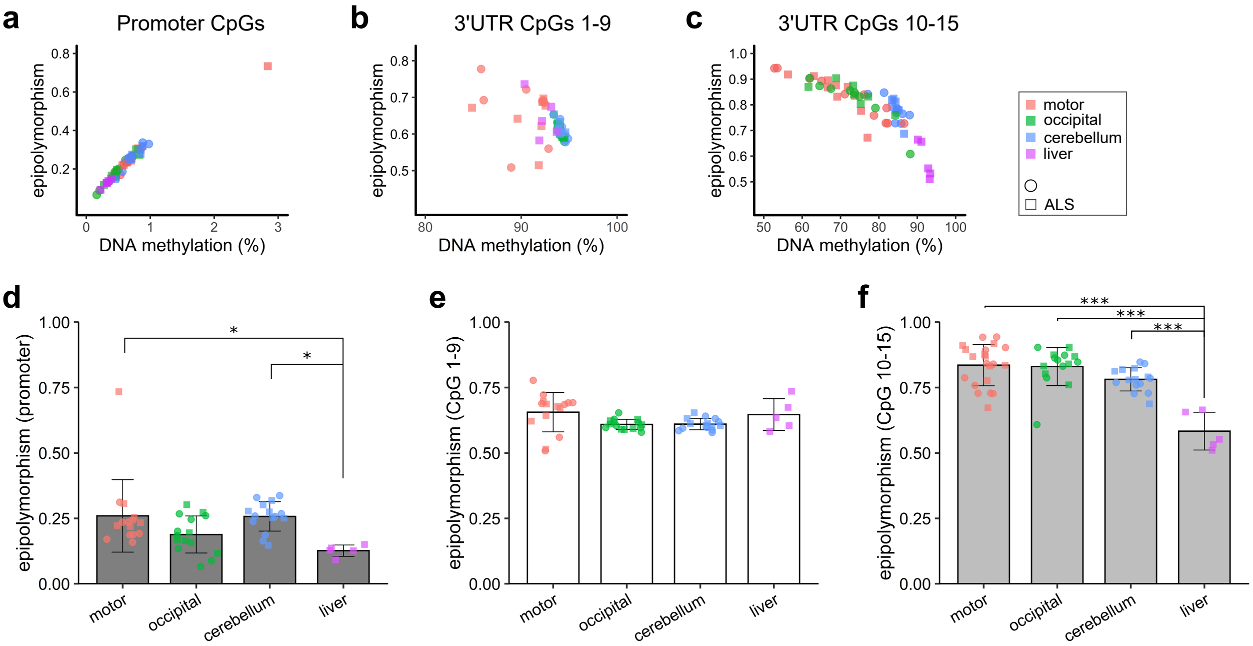

Supplementary Fig. 3 Epipolymorphism scores of each brain region and liver tissues. (a)-(c) Average DNA methylation percentages and epipolymorphism scores of promoter regions (a), 3'UTR CpGs 1–9 (b), and 3'UTR CpGs 10–15 (c). Epipolymorphism scores of promoter regions (d), 3'UTR CpGs 1–9 (e), and 3'UTR CpGs 10–15 (f) (mean±SD; Bonferroni's multiple comparison test). Ten ALS patients and 11 controls were included in the analysis of 3'UTR CpGs 10–15 in the motor cortex, five ALS patients were included in the analysis of the liver, and seven ALS patients and eight controls were included in the other analyses. ***p<0.001.

Supplementary Fig. 4

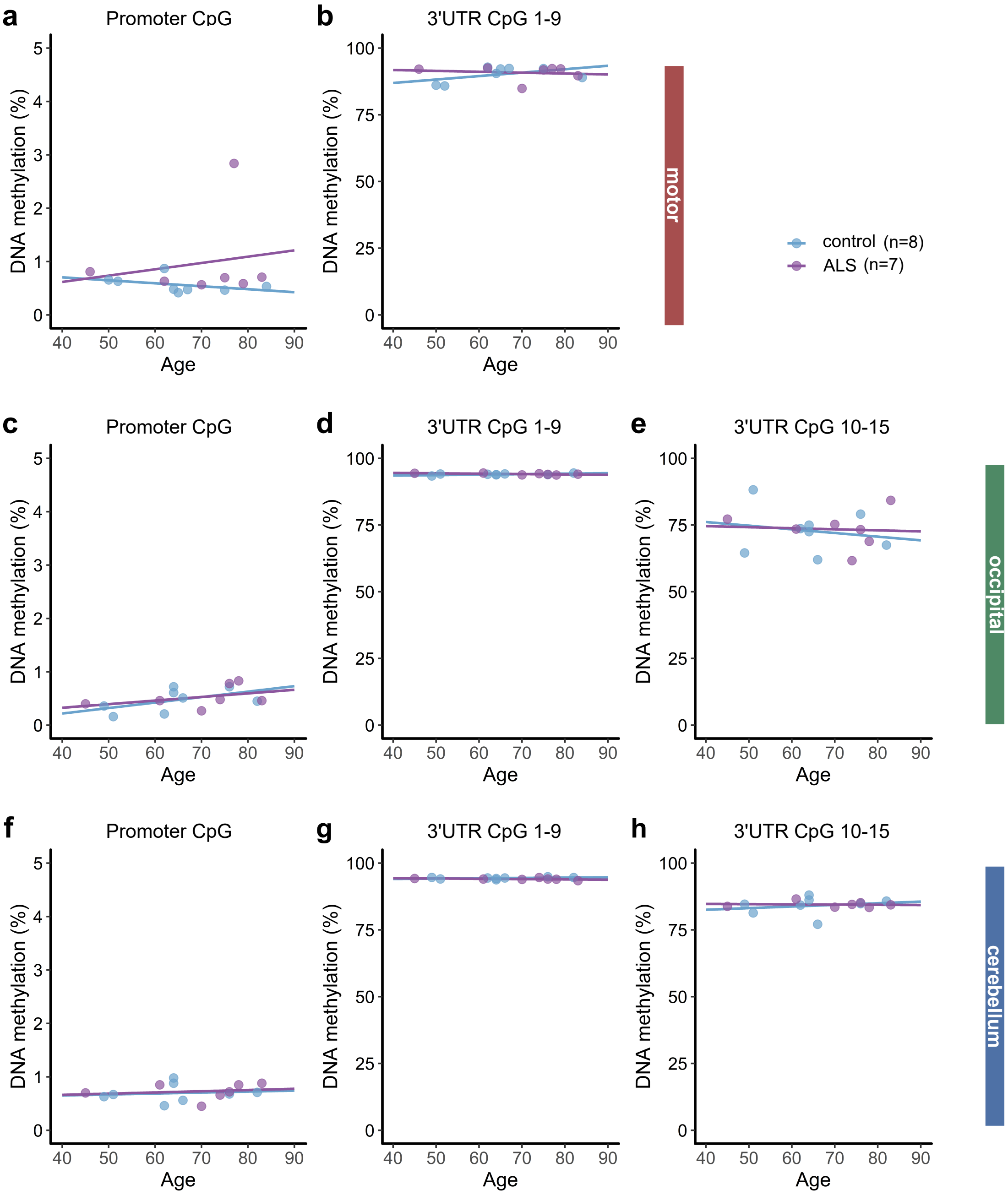

Supplementary Fig. 4 Scatter plot showing the DNA methylation of the TARDBP gene in the motor cortex (except for 3’UTR CpGs 10-15), occipital cortex and cerebellum according to age at autopsy. (a-h) Age at autopsy and mean DNA methylation percentages among promoter regions (a, c, f), 3'UTR CpGs 1–9 (b, d, g), and 3'UTR CpGs 10–15 (e, h). (a, b) Motor cortex, (c-e) occipital cortex, (f-h) cerebellum.

Supplementary Fig. 5

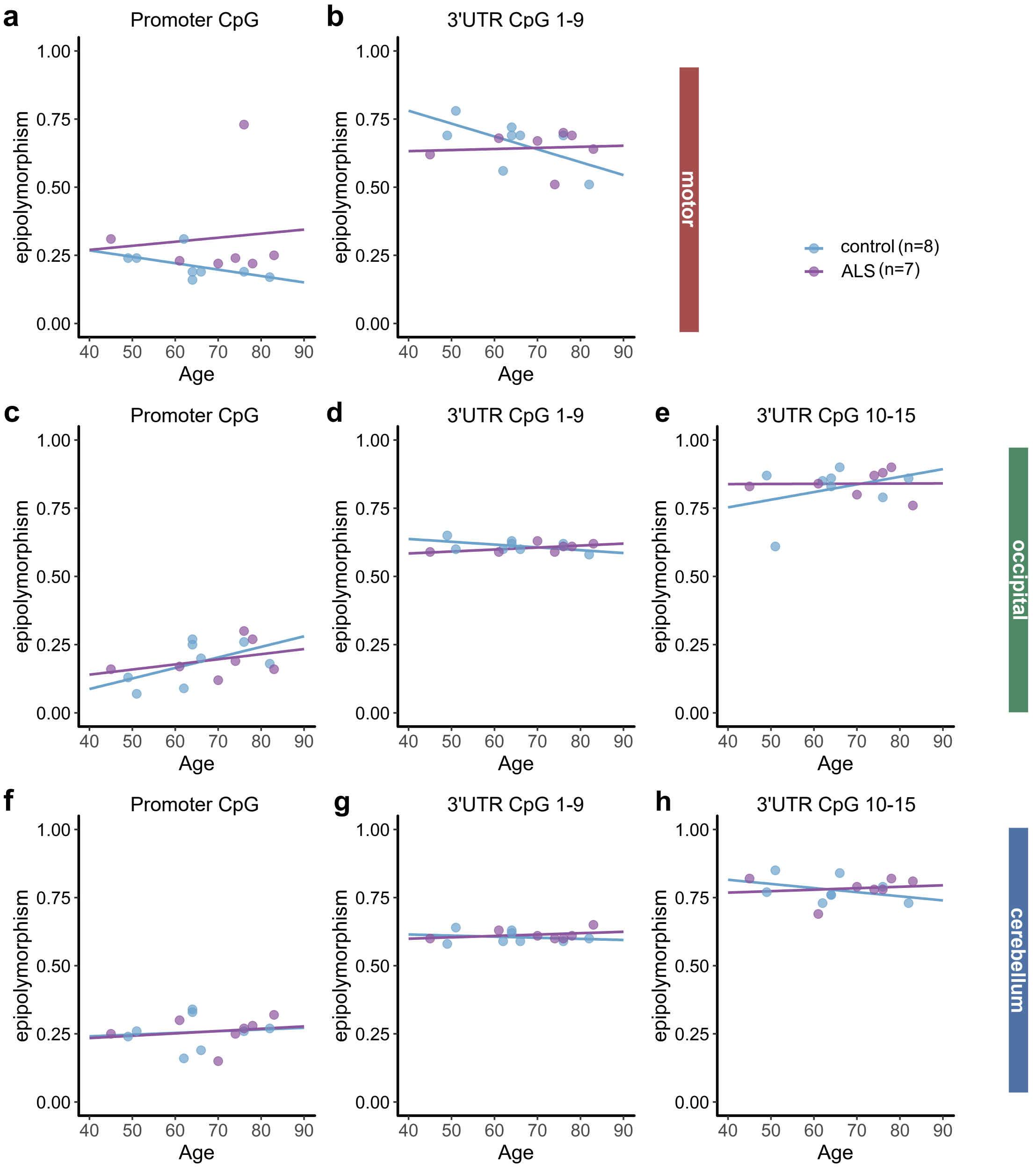

Supplementary Fig. 5 Scatter plot showing the epipolymorphism score of the TARDBP gene according to age at autopsy. (a-h) Age at autopsy and epipolymorphism scores of promoter regions (a, c, f), 3'UTR CpGs 1–9 (b, d, g), and 3'UTR CpGs 10–15 (e, h). (a-b) Motor cortex, (c-e) occipital cortex, and (f-h) cerebellum.

Supplementary Table 1. Clinical information of the postmortem cases

Case	Age	Sex	PMI (h:m)	Disease	Age of onset*	Disease duration* (month)	Initial symptom*	DNA methylation analysis				RNA analysis (RIN)	Western blotting
								motor cortex	occipital cortex	cerebellum	liver	motor cortex	motor cortex
ALS1	78	M	2:50	ALS	77	7	respiratory dysfunction	+	+	+	+	9.4	-
ALS2	61	F	4:00	ALS	57	46	upper limb	+	+	+	+	8.0	-
ALS3	76	M	1:30	ALS	74	20	bulbar	+	+	+	+	7.7	-
ALS4	45	F	6:30	ALS	44	17	upper limb + bulbar	+	+	+	+	6.2	-
ALS5	83	M	2:30	ALS	82	15	upper limb	+	+	+	+	8.7	-
ALS6	74	F	3:00	ALS	71	33	upper limb	+	+	+	-	-	+
ALS7	70	F	1:50	ALS	68	18	bulbar	+	+	+	-	8.9	+
ALS8	64	M	3:00	ALS	62	17	upper limb	+	-	-	-	-	+
ALS9	84	F	5:15	ALS	82	21	bulbar	+	-	-	-	6.7	+
ALS10	59	M	3:00	ALS	51	54	upper limb	+	-	-	-	8.3	+
control1	66	M	3:30	pellagra	-	-	-	+	+	+	-	-	+
control2	76	M	3:30	gastrointestinal bleeding	-	-	-	+	+	+	-	7.2	+
control3	64	M	2:00	renal failure	-	-	-	+	+	+	-	8.4	+
control4	64	F	2:00	polymyositis	-	-	-	+	+	+	-	9.1	+
control5	51	M	4:00	myopathy	-	-	-	+	+	+	-	7.3	+
control6	82	F	4:30	myasthenia gravis	-	-	-	+	+	+	-	7.7	+
control7	49	F	2:00	POEMS syndrome	-	-	-	+	+	+	-	9.0	+
control8	62	M	9:00	intermittent porphyria	-	-	-	+	+	+	-	-	+
control9	71	M	-	renal failure	-	-	-	+	-	-	-	-	+
control10	54	M	1:15	heart failure	-	-	-	+	-	-	-	8.0	+
control11	68	M	3:00	lung cancer	-	-	-	+	-	-	-	7.7	+

PMI (h:m): post mortem interval (hours: minutes), RIN: RNA integrity number
*Applicable to ALS cases only

Supplementary Table 2. Guide RNA target, PCR primer, and probe sequences

Name	Sequence (5' to 3')
guide RNA target sequence *TARDBP* 3'-UTR-1 (except PAM)	ATTCGTCATCACGCATCAC
guide RNA target sequence *TARDBP* 3'-UTR-2 (except PAM)	CATCTCATTTCAAATGTTTA
guide RNA target sequence *TARDBP* 3'-UTR-3 (except PAM)	CTCCTGTAATATTTTATCCC
guide RNA target sequence *TARDBP* 3'-UTR-4 (except PAM)	AATTCTTTGCATGTTCAAAA
TARDBP promoter primer set 1st F (bisulfite sequencing)	GTTGTTTTTTAGAAAAGGGGTTTAG
TARDBP promoter primer set 1st R (bisulfite sequencing)	AACTATATAAAAACTAACCTCCCCC
TARDBP promoter primer set 2nd F (bisulfite sequencing)	ACACTCTTTCCCTACACGACGCTCTTCCGATCTGGTTTTATTTTGTTTTTTAGGTGGATT
TARDBP promoter primer set 2nd R (bisulfite sequencing)	GTGACTGGAGTTCAGACGTGTGCTCTTCCGATCTGGAACTATATAAAAACTAACCTCCCCC
TARDBP 3'-UTR primer setA 1st F (bisulfite sequencing)	GTATTTTATTGAAAGTAGTGTTGTAAA
TARDBP 3'-UTR primer setA 1st R (bisulfite sequencing)	CACCATACAACATTCACAACAATTA
TARDBP 3'-UTR primer setA 2nd F (bisulfite sequencing)	ACACTCTTTCCCTACACGACGCTCTTCCGATCTGGTATAGGAATATTGTTTATATGTTTTTTT
TARDBP 3'-UTR primer setA 2nd R (bisulfite sequencing)	GTGACTGGAGTTCAGACGTGTGCTCTTCCGATCTGGCTTCTAATTCCATATCACAACCTTA
TARDBP 3'-UTR primer setB 1st F (bisulfite sequencing)	GTTGTGATATGGAATTAGAAGGTT
TARDBP 3'-UTR primer setB 1st R (bisulfite sequencing)	CACCATACAACATTCACAACAATTA (same as exon6-1 bisulfite 1st R)
TARDBP 3'-UTR primer setB 2nd F (bisulfite sequencing)	ACACTCTTTCCCTACACGACGCTCTTCCGATCTGGGTGTGGGATTGATGGTGGT
TARDBP 3'-UTR primer setB 2nd R (bisulfite sequencing)	GTGACTGGAGTTCAGACGTGTGCTCTTCCGATCTGGATTTCACTTAACACATTCATCTAAT
TARDBP F1 (RT-PCR)	GCGCTGTACAGAGGACATGA
TARDBP R1 (RT-PCR)	GCCTGTGATGCGTGATGA
TARDBP R2 (RT-PCR)	AGTTCCATCTCAAAAGGGTC
TARDBP unspliced F (qPCR)	TGTCACAGTGTTTGGTTCTTTTG
TARDBP unspliced R (qPCR)	AGCGGATAAAAATGGGACAC
RPLP1 F and R (qPCR, droplet digital PCR)	Purchased (Takara bio) primer set ID: HA067802
RPLP2 F and R (qPCR)	Purchased (Takara bio) primer set ID: HA067804
TARDBP mRNA F (droplet digital PCR)	CTGCGGGAGTTCTTCTCTCA
TARDBP mRNA R (droplet digital PCR)	CGCAATCTGATCATCTGCAA
TARDBP mRNA probe (droplet digital PCR)	CCCAAGCCATTCAGGGCCTTTG
RPLP1 probe (droplet digital PCR)	ACTGCTGCTGCTCCAGCTGA

Fig. 3b

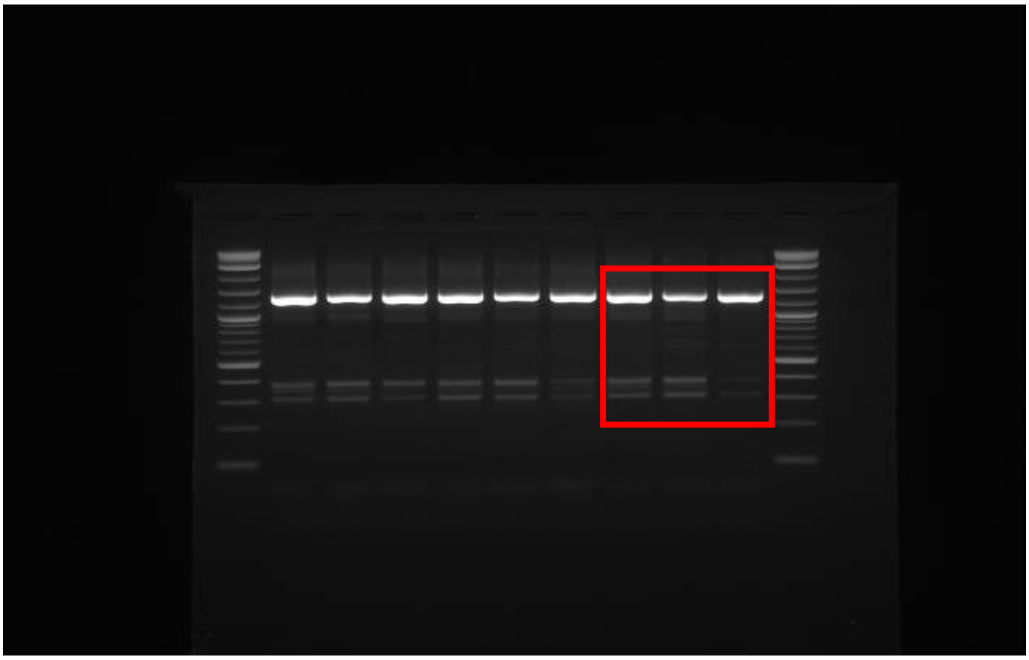

Fig. 3c

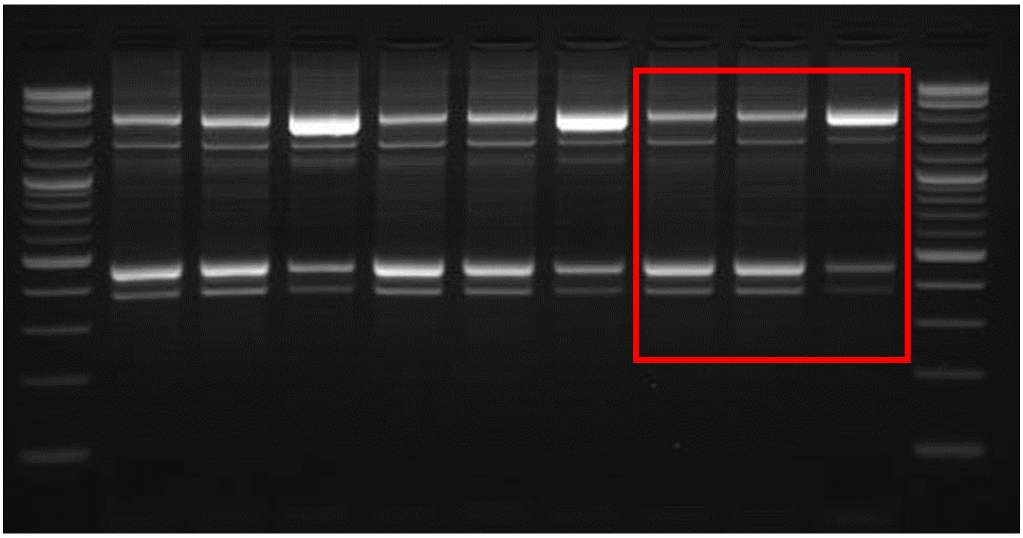

Fig. 7b

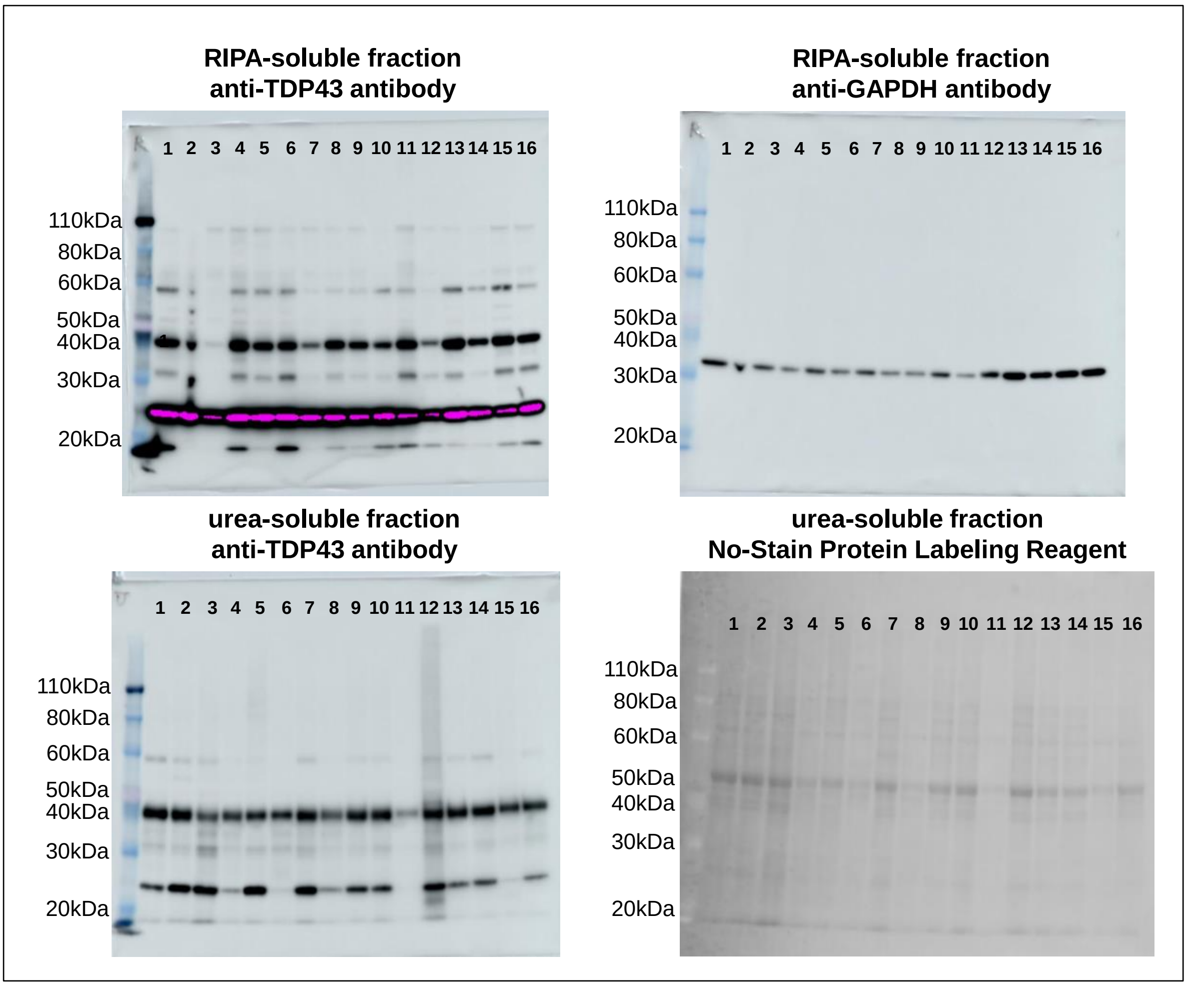
RIPA-soluble fraction
anti-TDP43 antibody
1 2 3 4 5 6 7 8 9 10 11 12 13 14 15 16
110kDa
80kDa
60kDa
50kDa
40kDa
30kDa
20kDa
RIPA-soluble fraction
anti-GAPDH antibody
1 2 3 4 5 6 7 8 9 10 11 12 13 14 15 16
110kDa
80kDa
60kDa
50kDa
40kDa
30kDa
20kDa
urea-soluble fraction
anti-TDP43 antibody
1 2 3 4 5 6 7 8 9 10 11 12 13 14 15 16
110kDa
80kDa
60kDa
50kDa
40kDa
30kDa
20kDa
urea-soluble fraction
No-Stain Protein Labeling Reagent
1 2 3 4 5 6 7 8 9 10 11 12 13 14 15 16
110kDa
80kDa
60kDa
50kDa
40kDa
30kDa
20kDa
