# Supplementary material for: Age-related demethylation of the TDP-43 autoregulatory region in the human motor cortex
Source: Commun Biol. 2021 Sep 21;4:1107. doi: 10.1038/s42003-021-02621-0 (PMC8455575; doi:10.1038/s42003-021-02621-0)
Supplement: Supplementary file 3 — Description of Additional Supplementary Files [file 42003_2021_2621_MOESM3_ESM.pdf]

Description of Additional Supplementary Files

File name: Supplementary Data 1.
Description: Summarized excel file containing the original information for all experiment-based figures and supplementary figures in this paper.
