# Supplementary material for: Age-related demethylation of the TDP-43 autoregulatory region in the human motor cortex
Source: Commun Biol. 2021 Sep 21;4:1107. doi: 10.1038/s42003-021-02621-0 (PMC8455575; doi:10.1038/s42003-021-02621-0)
Supplement: Supplementary file 5 — Reporting Summary [file 42003_2021_2621_MOESM5_ESM.pdf]

nature research

Corresponding author(s): Osamu Onodera

Last updated by author(s): Aug 13, 2021

Reporting Summary

Nature Research wishes to improve the reproducibility of the work that we publish. This form provides structure for consistency and transparency in reporting. For further information on Nature Research policies, see our Editorial Policies and the Editorial Policy Checklist.

Statistics

For all statistical analyses, confirm that the following items are present in the figure legend, table legend, main text, or Methods section.

n/a	Confirmed	
☐	☒	The exact sample size (*n*) for each experimental group/condition, given as a discrete number and unit of measurement
☐	☒	A statement on whether measurements were taken from distinct samples or whether the same sample was measured repeatedly
☐	☒	The statistical test(s) used AND whether they are one- or two-sided<br>*Only common tests should be described solely by name; describe more complex techniques in the Methods section.*
☐	☒	A description of all covariates tested
☐	☒	A description of any assumptions or corrections, such as tests of normality and adjustment for multiple comparisons
☐	☒	A full description of the statistical parameters including central tendency (e.g. means) or other basic estimates (e.g. regression coefficient) AND variation (e.g. standard deviation) or associated estimates of uncertainty (e.g. confidence intervals)
☒	☐	For null hypothesis testing, the test statistic (e.g. F, t, r) with confidence intervals, effect sizes, degrees of freedom and P value noted<br>*Give P values as exact values whenever suitable.*
☒	☐	For Bayesian analysis, information on the choice of priors and Markov chain Monte Carlo settings
☒	☐	For hierarchical and complex designs, identification of the appropriate level for tests and full reporting of outcomes
☒	☐	Estimates of effect sizes (e.g. Cohen's *d*, Pearson's *r*), indicating how they were calculated

Our web collection on statistics for biologists contains articles on many of the points above.

Software and code

Policy information about availability of computer code

Data collection	Cell sorting: FACS Diva (BD) was used on FACSAria II for isolating GFP-positive HEK293T cells.<br><br>DNA methylation analyses: Illumina Miseq Control software (3.1) was used on the illumina Miseq sequencers to collect the sequencing data. The sequenced reads were mapped to the human reference genome hg19 using the methylation analysis tool Bismark v0.18.1 (Babraham Bioinformatics). Then, the pileups of the sequence data at targeted CpG sites were generated using GATK's pileup command.<br><br>RNA analyses of HEK293T cells: For RT-PCR, the quantification of each band on gel images was performed using Image Quant TL analysis software (GE Healthcare). Quantitative real-time PCR was performed on the TP-850 Real-Time PCR Detection System (TAKARA Bio).<br><br>RNA analysis of the human motor cortex: Droplet digital PCR was performed on the QX 200 Droplet Digital PCR System (Bio Rad).<br><br>Western blot analysis of the human motor cortex: The quantitative analysis for western blot was performed by Amersham Imager 680 (GE Healthcare).
Data analysis	R package v3.5.1 was used for statistics and visualization of the data. The details are as follows: ggplot2 package was used to make graphs; 'heatmap.2' gplots package was used to make heatmaps; 'pcaMethods' R package was used for the principal component analysis.

For manuscripts utilizing custom algorithms or software that are central to the research but not yet described in published literature, software must be made available to editors and reviewers. We strongly encourage code deposition in a community repository (e.g. GitHub). See the Nature Research guidelines for submitting code & software for further information.

Data

Policy information about availability of data

All manuscripts must include a data availability statement. This statement should provide the following information, where applicable:

- Accession codes, unique identifiers, or web links for publicly available datasets
- A list of figures that have associated raw data
- A description of any restrictions on data availability

Source data for Fig.1-8, Supplemental Fig.1-5, and Supplemental Table1-2 are available with the paper. All other data are available from the corresponding authors upon reasonable request.

Field-specific reporting

Please select the one below that is the best fit for your research. If you are not sure, read the appropriate sections before making your selection.

☒ Life sciences ☐ Behavioural & social sciences ☐ Ecological, evolutionary & environmental sciences

For a reference copy of the document with all sections, see nature.com/documents/nr-reporting-summary-flat.pdf

Life sciences study design

All studies must disclose on these points even when the disclosure is negative.

Sample size	In the analyses of human tissues, we estimated an effect size of 0.8-0.9 in the human motor cortex and calculated the sample size by setting the power to 0.8 and the significance level to 0.05 based on the effect sizes in the correlation analyses in the previous related reports. In the experiment manipulating DNA methylation status, sample sizes were chosen based on prior literature using similar experimental paradigms.
Data exclusions	No data was excluded.
Replication	All experiments were repeated at least once. All attempts at replication were successful.
Randomization	No randomization was performed.
Blinding	Blinding was not performed in these analyses.

Reporting for specific materials, systems and methods

We require information from authors about some types of materials, experimental systems and methods used in many studies. Here, indicate whether each material, system or method listed is relevant to your study. If you are not sure if a list item applies to your research, read the appropriate section before selecting a response.

Materials & experimental systems

n/a	Involved in the study	
☒	☐	Antibodies
☐	☒	Eukaryotic cell lines
☒	☐	Palaeontology and archaeology
☒	☐	Animals and other organisms
☐	☒	Human research participants
☒	☐	Clinical data
☒	☐	Dual use research of concern

Methods

n/a	Involved in the study	
☒	☐	ChIP-seq
☐	☒	Flow cytometry
☒	☐	MRI-based neuroimaging

Eukaryotic cell lines

Policy information about cell lines

Cell line source(s)	HEK293T cells
Authentication	Cell line authentication was performed by the supplier, but not independently authenticated in our lab.
Mycoplasma contamination	Cell lines tested negative for myoplasma.
Commonly misidentified lines (See ICLAC register)	No commonly misidentified lines were used.

Human research participants

Policy information about studies involving human research participants

Population characteristics	All human tissues were provided by the Department of Pathology, Brain Research Institute, Niigata University. The Institutional Ethical Review Board of Niigata University approved this study, which investigated postmortem tissues autopsied with written informed consent from families.
Recruitment	N/A
Ethics oversight	N/A

Note that full information on the approval of the study protocol must also be provided in the manuscript.

Flow Cytometry

Plots

Confirm that:

- [ ] The axis labels state the marker and fluorochrome used (e.g. CD4-FITC).
- [ ] The axis scales are clearly visible. Include numbers along axes only for bottom left plot of group (a 'group' is an analysis of identical markers).
- [ ] All plots are contour plots with outliers or pseudocolor plots.
- [ ] A numerical value for number of cells or percentage (with statistics) is provided.

Methodology

Sample preparation	HEK293T cells were collected and resuspended in Dulbecco's Modified Eagle's Medium containing 10% fetal bovine serum.
Instrument	Cell sort: FACSAria II (BD)
Software	FACS Diva (BD)
Cell population abundance	250,000 GFP-positive cells per each sample were collected.
Gating strategy	Gates were set based on a negative control sample that had no treatment.<br>The plots exemplifying the gating strategy are not on record and cannot be provided.

- [ ] Tick this box to confirm that a figure exemplifying the gating strategy is provided in the Supplementary Information.
